# Cognitive behavioural therapy on improving the depression symptoms in patients with diabetes: a meta-analysis of randomized control trials

**DOI:** 10.1042/BSR20160557

**Published:** 2017-04-10

**Authors:** Zhi-da Wang, Yu-fei Xia, Yue Zhao, Li-ming Chen

**Affiliations:** 1 Key Laboratory of Hormones and Development (Ministry of Health), Tianjin Key Laboratory of Metabolic Diseases, Tianjin Metabolic Diseases Hospital & Tianjin Institute of Endocrinology, Tianjin Medical University, Tianjin 300070, China; 2 School of Nursing, Tianjin Medical University, Tianjin 300070, China

**Keywords:** cognitive behavioral therapy, depression, diabetes, meta analysis

## Abstract

This meta-analysis was performed to evaluate the effect of cognitive behavioural therapy (CBT) in improving the depression symptoms of patients with diabetes. Literature search was conducted in PubMed and Embase up to October 2016 without the initial date. The pooled SMD (standard mean difference) and its 95% confidence interval (CI) were calculated by Revman 5.3. Subgroup analyses were performed by type of diabetes and evaluation criteria of depression. A total of five randomized control trials involving 834 patients with diabetes mellitus (including 417 patients in CBT group and 417 patients in control group) were included in this meta-analysis. The pooled estimates indicated significant improvement of depression by CBT compared with routine approaches in overall outcomes (SMD =–0.33, 95% CI =–0.46 to –0.21, *P*<0.00001), post-intervention outcomes (SMD =–0.43, 95% CI =–0.73 to –0.12, *P*=0.006) and outcomes after 12 months intervention (SMD =–0.38, 95% CI = –0.54 to –0.23, *P*<0.0001). Subgroup analyses showed that the results were not influenced by the type of diabetes. However, the effect of CBT on improving the depression symptoms disappeared when only using CES-D (Centre for Epidemiological Studies scale for Depression) to evaluate depression.

## Introduction

Both diabetes and depression were highly prevalent health problems and there was interrelationship between diabetes and depression [1]. Approximately 20–30% of patients with diabetes suffered from depressive disorders, which was more common than among the general population [2,3]. Meanwhile, depression was found to be associated with the increased risk of diabetes mellitus [4] and higher risk of complications and adverse outcomes in patients with diabetes mellitus [5,6]. Moreover, treatment non-adherence caused by depression [7] could result in poorer self-care behaviour [8,9]. Thus, the improvement of depression was important for the patients with diabetes mellitus.

Cognitive behavioural therapy (CBT) was a well-known psychological therapy for effectively challenging dysfunctional thoughts, beliefs and negative behaviours. The efficacy of CBT on anxiety and depression has been proved in many recent studies [10–12]. Currently, many studies also investigated the effect of CBT on improving the depression symptoms in patients with diabetes [13–15]. Although a recent meta-analysis has proved the effectiveness of CBT on depression in patients with diabetes [16], evidence was not strong enough due to some limitations. Firstly, not all relevant studies were included in this previous meta-analysis [17–19]. Secondly, some studies with high risk of attrition bias (which reported incomplete outcome data) were used and analyzed in that previous meta-analysis [20–22]. Thus, we performed this meta-analysis to further evaluate the effect of CBT on depression symptoms in patients with diabetes by studies with low risk of attrition bias. Moreover, the impact of type of diabetes and evaluation criteria of depression on results was assessed by subgroup analyses in the present study.

## Materials and methods

### Literature search strategy

A literature search was conducted in PubMed and Embase databases without initial publication date and with the deadline of October 2016, using the following keywords: ‘diabetes’ AND ‘depression’ AND ‘Cognitive Behaviour’ OR ‘Cognitive Behavioural’ AND ‘Randomized’, with language limitation in English. Additional articles were searched by reviewing the references of relevant studies.

### Inclusion and exclusion criteria

All included studies should meet the following criteria: (i) study type should be randomized control trial (RCT); (ii) participants were patients with diabetes mellitus aged older than 18 years; (iii) the effect of CBT (CBT group) on the depression symptoms was evaluated by comparing with usual care or other routine therapies (control group) in patients with diabetes mellitus; (iv) the outcomes of depression symptoms were evaluated.

Exclusion criteria were: (i) duplicated publications (only the study with most complete data were included), (ii) letters, comments, case reports or reviews and (iii) no available data.

### Data extraction and quality assessment

Two investigators independently reviewed the full texts of included studies and assessed their quality. Differences were resolved by discussion to ensure consistency of evaluation. The methodological quality of these randomized trials was assessed using the Cochrane Collaboration’s tool for assessing risk of bias [23], which included eight items: random sequence generation, allocation concealment, blinding of participants and personnel, blinding of outcome assessment, assessment of incomplete data outcome, selective reporting and other sources of bias.

The following data should be extracted using a predesigned form: first author name, year of publication, country, duration of intervention, follow-up duration, control group, sample size, age, sex, evaluation criteria of depression, the baseline and outcomes.

### Statistical analysis

In this meta-analysis, Reviewer manager 5.3 was used to calculate the pooled SMD (standard mean difference) as well as the corresponding 95% confidence interval (CI). The Chi-square and *I*-square statistic were used to assess the between-studies heterogeneity. A *P* value <0.1 or I^2^>50% indicated the significant heterogeneity, then, the random-effect model was used to pool the SMDs. Otherwise, the fixed-effect model was applied. A Z-test was used to determine the statistical significance of these pooled SMDs. In addition, subgroup analyses were performed by type of diabetes or the evaluation criteria of depression. Publication bias was assessed by Egger’s test using Stata 11.0. The trim-and-fill method was used to estimate the results of adjusted meta-analysis when publication bias presented. For all these analysis, the *P* value of <0.05 indicated statistical significance.

## Results

### Characteristics of included studies

**Figure 1:**
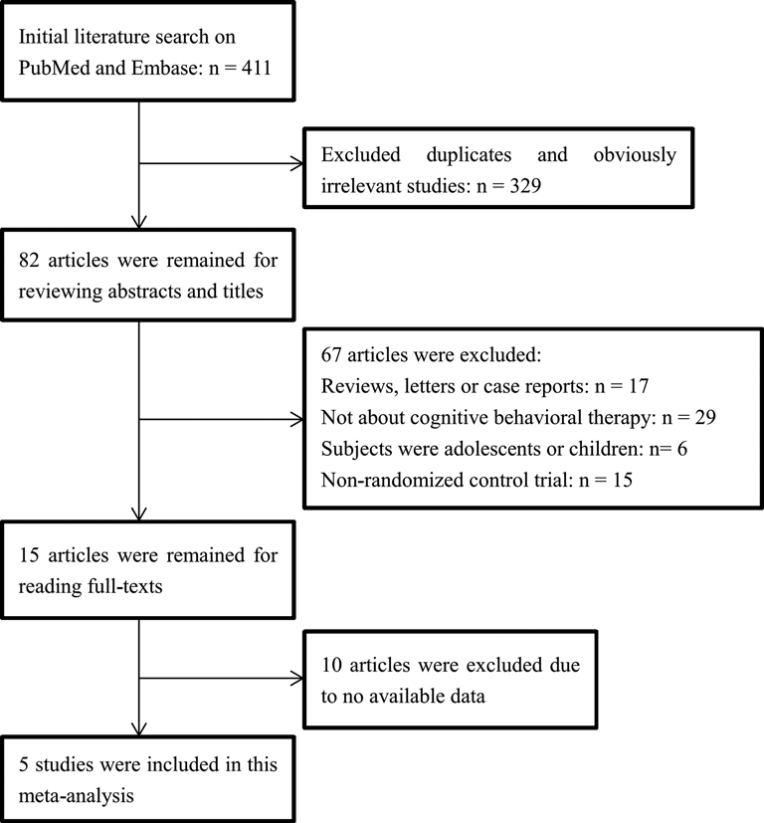
Flow diagram of the study selection process

The flow chart of literature search and study selection was presented in Figure 1. After initial literature search, a total of 411 articles were identified from PubMed and Embase. After excluding duplicates and irrelevant articles, 82 potentially relevant articles remained. Among them, 67 articles were removed by scanning the titles or abstracts: 17 non-original articles (reviews, letters or case reports), 29 articles not about CBT, 6 articles on adolescents or children and 15 non-RCTs, according to the inclusion and exclusion criteria. After reading the full-text, ten articles without available data were excluded. Finally, five studies were included in this meta-analysis [17–19,24,25].

**Figure 2:**
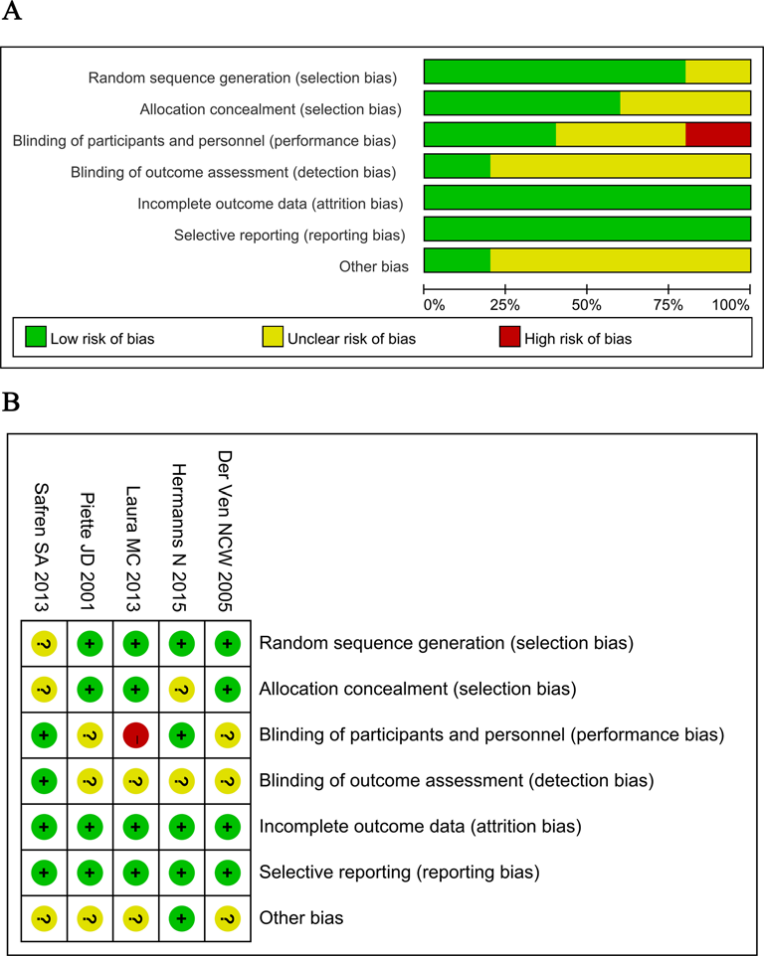
Quality assessment of included studies: risk of bias graph (**A**) and risk of bias summary (**B**)

The characteristics of these included studies were listed in Table 1. A total of five studies involving 834 patients with diabetes mellitus (including 417 patients in CBT group and 417 patients in control group) were included in this meta-analysis. The publication year ranged from 2005 to 2015. The intervention duration was reported in three studies [18,24,25] and ranged from 3 to 12 months. Three studies reported the outcomes after 12 months follow up [17,19,25]. There were no significant differences in age and sex between groups in these included studies. The criteria for evaluating depression were CES-D (Centre for Epidemiological Studies scale for Depression), BDI (Beck Depression Inventory), MADRS (Montgomery–Asberg Depression Rating Scale), CGI (Clinical Global Impression) or/and PHQ-9 (Patient Health Questionnaire-9) in these studies. The basic values of these criteria were similar between CBT and control groups in these included studies. In addition, Figure 2 showed that there was no high risk of bias in all these included RCTs, except the study of Laura MC et al. [19], which showed high risk of performance bias because patients were not blinded to interventions (Figure 2).

**Table 1:** Characteristics of these included studies

Author (year)	Country	Duration of intervention	Follow-up duration	Subjects	Group	Sample size	M/F	Age	Criteria for evaluating depression	Baseline values
der Ven, N.C.V. (2005)	Netherlands	3 months	0 months	Type 1 diabetes	CBT	45	36/52	37.8 ± 10.6	CES-D	16.0 ± 11.0
					Control: blood glucose awareness training	43				
Piette, J.D. (2011)	U.S.A.	12 months	0 months	Type 2 diabetes	CBT	145	72/73	55.1 ± 9.4	BDI	26.7 ± 7.7
					Control: usual care	146	73/73	56.0 ± 10.9		26.5 ± 9.9
Safren, S.A. (2014)	U.S.A.	4 months	12 months	Type 2 diabetes	CBT	45	22/23	55.44 ± 8.72	MADRS	25.60 ± 8.99
									CGI	4.42 ± 1.29
					Control: enhanced treatment as usual	42	22/20	58.31 ± 7.41	MADRS	23.31 ± 7.20
									CGI	3.98 ± 1.09
Laura, M.C. (2013)	Netherlands	NA	12 months	Type 2 diabetes	CBT	76	45/31	60.5 ± 9.4	CES-D	11.1 ± 8.1
					Control: managed care	78	50/28	61.2 ± 8.8		9.6 ± 8.2
Hermanns, N. (2015)	Germany	NA	12 months	Diabetes mellitus	CBT	106	56/60	43.2 ± 14.9	CES-D	24.7 ± 7.6
									PHQ-9	10.9 ± 4.3
					Control: diabetes education	108	57/61	43.4 ± 13.8	CES-D	22.4 ± 8.6
									PHQ-9	9.6 ± 3.8

### Overall outcomes

**Figure 3:**
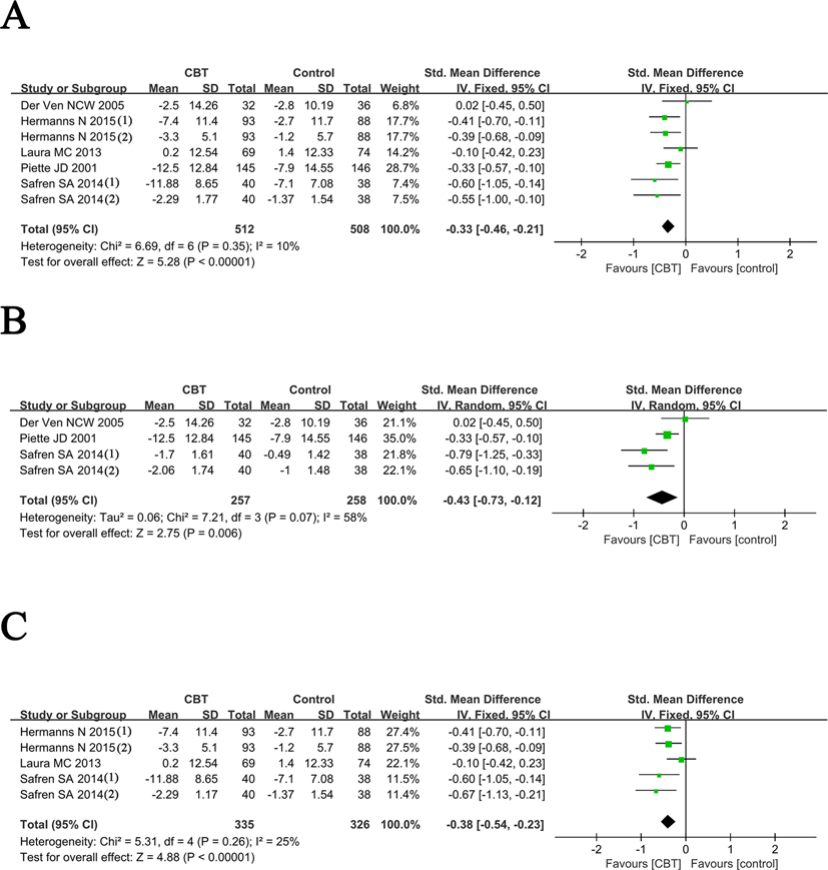
Forest plots for meta-analysis of overall outcomes (**A**), post-intervention outcomes (**B**) and outcomes after 12 months follow up (**C**) Hermanns, N. 2015 (i): outcomes of depression evaluated by CES-D; Hermanns, N. 2015 (ii): outcomes of depression evaluated by PHQ-9; Safren, S.A. 2013 (I): outcomes of depression evaluated by MADRS; Safren, S.A. 2013 (II): outcomes of depression evaluated by CGI.

As shown in Figure 3A, we re-analyzed the mean change of depression symptoms (mean difference of depression score from endpoint to baseline) between CBT and control groups. The results showed there was no significant heterogeneity (I^2^ = 10%, *P*=0.35) among these included studies, so the fixed-effect model was used. The pooled estimate indicated that the depression symptom of diabetes mellitus patients in CBT group was significantly improved compared with that in the control group.

### Meta-analysis for post-intervention outcomes

Three studies [18,24,25] analyzed the mean change of depression symptoms after intervention immediately (mean difference of depression score from post-intervention to baseline) between CBT and control groups. Significant heterogeneity among these studies (I^2^ =58%, *P*=0.07) was found, so the random-effect model was used. The pooled SMD was –0.43 (95% CI =–0.73 to –0.12, Z =2.75, *P*=0.006), indicating better efficacy of CBT in reducing depression symptoms than the controls at immediate post-intervention (Figure 3B).

### Meta-analysis for outcomes after 12-months follow up

The mean change of depression symptoms after 12 months follow up (mean difference of depression score from 12 months follow up to baseline) between CBT and control groups was analyzed in three included studies [17,19,25]. No significant heterogeneity (I^2^ =25%, *P*=0.26) was found among these studies, then we applied the fixed-effect model to pool the data. The pooled estimate (SMD =–0.38, 95% CI =–0.54 to –0.23, Z =4.88, *P*<0.0001) indicated that the depression symptoms still be significantly improved by CBT after 12 months follow up, compared with controls.

### Subgroup analyses

Table 2 showed the results of subgroup analyses.

**Table 2:** Results of subgroup analyses

Subgroup analyses		Number of included studies	SMD [95% CI]	*P* value	Heterogeneity (I^2^, *P* value)
Analyses based on studies only involving Type 2 diabetes patients	Overall analysis	3 [19,24,25]	–0.34 [–0.50, –0.17]	<0.0001	28%, *P*=0.24
	Post-intervention outcomes	2 [24,25]	–0.53 [–0.82, –0.24]	0.0004	46%, *P*=0.16
	Outcomes after 12 months follow up	2 [19,25]	–0.43 [–0.81, –0.04]	0.03	62%, *P*=0.07
Analyses based on studies in which depression was evaluated using CES-D	Overall analysis	3 [17–19]	–0.21 [–0.56, 0.13]	0.23	52%, *P*=0.12
	Post-intervention outcomes	1 [18]	0.02 [–0.45, 0.50]	0.92	-
	Outcomes after 12 months follow up	2 [17,19]	–0.32 [–0.81, 0.17]	0.20	67%, *P*=0.08

-, no application.

For the analyses on studies only enrolled patients with Type 2 diabetes, the significant heterogeneity existed among studies for the outcomes after 12 months follow up (*P*=0.07, I^2^ =62%), so the randomized-effect model was used. No significant heterogeneity was found for overall outcomes and post-intervention outcomes (*P*>0.10, I^2^ <50%), and then the fixed-effect model was applied. The results of subgroup analyses showed consistent results with the overall analyses, indicating that the efficacy of CBT on depression symptoms was not affected by the type of diabetes. In addition, the disappearance of heterogeneity in analysis for post-intervention outcomes in subgroup analyses indicated that the type of diabetes may be one of the sources of heterogeneity among studies (Table 2).

For the analyses on studies in which depression was evaluated using CES-D, the significant heterogeneity among studies was found in analyses for overall outcomes and outcomes after 12 months follow up (*P*<0.10, I^2^ >50%), so the random-effect model was used. However, inconsistent result with the overall analysis was found in these subgroup analyses, indicating the similar mean change of CES-D score between CBT and control groups (Table 2). These results suggested that the results can be affected by the evaluation criteria of depression.

### Publication bias

The significant publication bias did not exist in this meta-analysis based on the results of the Egger’s test (overall outcomes: *P*=0.732, post-intervention outcomes: *P*=0.372, outcomes after 12 months follow up: *P*=0.975).

## Discussion

The present study proved the effectiveness of CBT on improving depression after intervention immediately and 12 months follow up in patients with diabetes, compared with controls. However, the effectiveness of CBT on depression symptoms was similar to the controls when only using CES-D to evaluate depression.

Compared with the recent meta-analysis [16], we excluded the studies of Amsberg et al. [22] and Snoek et al. [21] because of incomplete outcome data and high risk of attrition bias. In addition, the studies of der Ven et al. [18] and Hermanns et al. [17] were first analyzed in this meta-analysis. Furthermore, we evaluate the efficacy of CBT on depression at post-intervention and 12 months after follow up. In addition, we performed the subgroup analyses and found the impact of evaluation criteria of depression on results, which was not shown in the previous meta-analysis [16].

As we all know that depression causing low medication adherence was always a major problem in the treatment of diabetes [9,26,27]. CBT can improve the medication adherence in many diseases including diabetes, which may be via reducing the depression symptoms [28,29]. In addition, previous studies also found the association between depression and glycaemic control in patients with diabetes [30,31]. Some evidence have proved the efficacy of treatments for depression on improving the glycaemic control in patients with diabetes [32,33]. Thus, glycaemic control may be associated with the mechanism of improved depression symptoms by CBT in patients with diabetes [16].

Although the overall analysis confirmed the effectiveness of CBT on improving depression symptoms of patients with diabetes compared with the routine approaches, the impact of evaluation criteria of depression on results should not be ignored. The CES-D was a short self-report scale designed to measure depressive symptomatology in the general population [34]. Although the studies have showed that CES-D was a valid screening tool for depression in diabetic patients, the sensitivity and specificity was not high enough to discriminate depression [35–37]. Thus, the CES-D may be inappropriate to evaluate the efficacy of CBT on improving depression in patients with diabetes, then resulting in the non-significant results.

Some limitations of this meta-analysis should be noted. Firstly, these evidences from this meta-analysis were not strong enough due to the small sample size. More studies with larger sample size should be performed to verify these results. Secondly, although the type of diabetes may be one of the sources of heterogeneity among studies, significant heterogeneity among studies was still present, which may be caused by the other confounding factors (such as intervention duration and controls). Thirdly, long follow up should be performed in future studies to not only assess the depression outcomes but also the other outcomes such as medication adherence and glycaemic control. Furthermore, except the three criteria analyzed in this meta-analysis, there was many other criteria for evaluating depression such as ‘beck depression inventory’, ‘zung depression rating scale’ or ‘hospital anxiety and depression scale’. However, there were not enough studies considering these criteria, so this meta-analysis could not analyze them due to lack of available data.

In conclusion, CBT had better efficacy in improving depression symptoms of patients with diabetes compared with routine approaches.

## Abbreviations

BDI: Beck depression inventory
CBT: cognitive behavioural therapy
CES-D: Centre for Epidemiological Studies scale for Depression
CGI: clinical global impression
CI: confidence interval
MADRS: Montgomery–Asberg depression rating scale
PHQ-9: patient health questionnaire-9
RCT: randomized control trial
SMD: standard mean difference
